# HER2/neu-based vaccination with li-Key hybrid, GM-CSF immunoadjuvant and trastuzumab as a potent triple-negative breast cancer treatment

**DOI:** 10.1007/s00432-023-04574-9

**Published:** 2023-01-24

**Authors:** Yihan Zhou

**Affiliations:** Department of Oncology, Medical Sciences Division, University of Oxford, Oxford, UK

**Keywords:** HER2/neu-based vaccine, Clinical trials, Triple-negative breast cancer, Breast cancer treatment

## Abstract

**Purpose:**

Constituting 15 to 20% of breast cancer cases, the triple-negative subtype lacks effective treatments as being less responsive to hormone-associated therapies. Alternatively, a more powerful immunotherapeutic vaccination can trigger immune recognition and destruction against breast cancer by incorporating oncological antigens such as human epidermal growth factor receptor 2 (HER2/neu). Currently, HER2/neu-based vaccines have finished three phases with breast cancer patients, in conjunction with granulocyte-macrophage colony-stimulating factor (GM-CSF) that was proven to be a promising vaccine adjuvant in other cancer trials previously.

**Methods:**

Completed HER2/neu-based vaccine trials with GM-CSF immunoadjuvants for breast cancer were summarised, and additionally, the article discussed prominent findings of vaccine effectiveness in triple-negative breast cancer, regarding li-Key hybrid in vaccine design and co-administration of anti-HER2/neu trastuzumab.

**Results:**

Nine clinical trials of three HER2/neu epitopes, one with li-Key hybrid, were analysed with or without the presence of trastuzumab. Immunological responses and minimal toxicities were observed in these epitopes, and disease-free survival was especially improved in the triple-negative population.

**Conclusion:**

HER2/neu-based peptide vaccine is a safe and effective approach against breast cancer, and its benefits can be potentially furthered by combining the li-Key hybrid vaccine with targeted drugs and adjuvants selected to enhance cross-presentation for exogenous vaccine antigens.

**Graphical abstract:**

**Figure:**
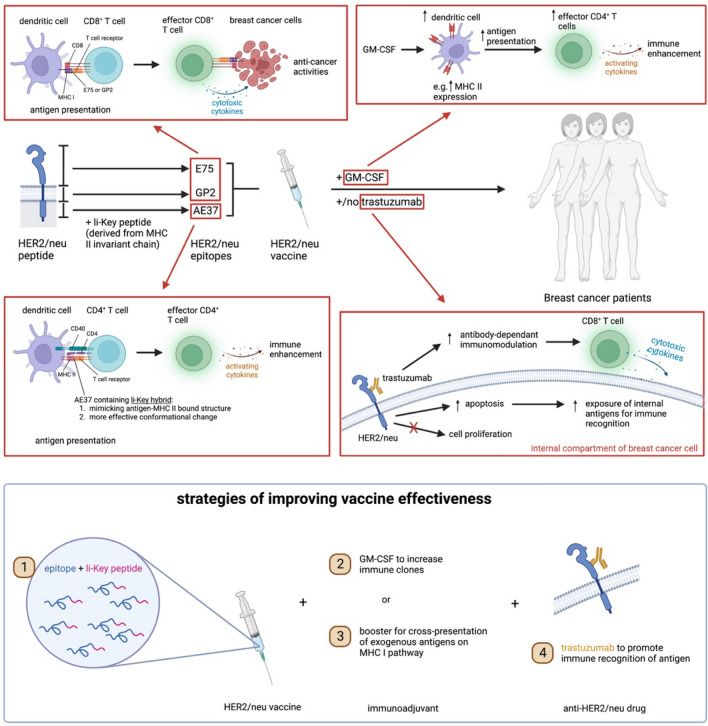
Graphical abstract was created with Biorender.com (license number: HA24UHRBV4 and FP24UHRGDD)

## Introduction

Female breast cancer is the most frequent malignancy and ranked as the fifth cause of mortality among all cancer types worldwide. According to the World Health Organisation (WHO) Global Cancer (GLOBOCAN) Statistics (Sung et al. 2021), breast cancer accounted for 2.26 million new cases diagnosed in 2020 and over 600,000 deaths, with an estimation of the incidence of 4.3 million new cases and 1.4 million deaths by 2040 (Ferlay et al. 2020). Among all subtypes of breast cancer, triple-negative breast cancer is responsible for 15 to 20% of breast cancer, and is the most aggressive for poor therapeutic prognosis due to its major characteristic of the lack of expression of human epidermal growth factor receptor 2 (HER2/neu), oestrogen receptor and progesterone receptor (Smolarz et al. 2022). In this case, therapies that target these three hormone receptors or hormonal therapy are less effective, as the pathogenesis of triple-negative breast cancer is rather independent of hormone-associated dysregulation. Developing a safe and novel therapy has drawn massive research attention, to overcome the healthcare burden and epidemiological severity of triple-negative breast cancer.

In the advance to all current cancer treatments, the emergence of immunotherapy has constructed a promising therapeutic approach and revolutionised the treatment development for cancer. As an active immunotherapeutic approach, a therapeutic cancer vaccine is designed to involve immune or tumour cells, tumour-associated peptides or genetic features. Most vaccines for breast cancer usually apply the strategies of utilising antigenic peptides that help immune cells to differentiate breast cancer cells from normal cells, subsequently initiating breast cancer-specific immunity (Abbaspour and Akbari 2022). Meanwhile, vaccines were co-administrated with adjuvants, which aim to further enhance the immune response against the vaccine target, and thereby, improve the anti-cancer efficacy of vaccination (Liu et al. 2021). As an example, HER2/neu peptide, which has addressed the pathological relevance to breast cancer, has been developed as the breast cancer vaccine and is currently assessed under ongoing clinical trials with the immunoadjuvant, granulocyte–macrophage colony-stimulating factor (GM-CSF) (Abbaspour and Akbari 2022). Therefore, this article will elucidate the fundamental therapeutic mechanism behind peptide-based cancer vaccination for breast cancer, HER2/neu-based vaccination using GM-CSF as the adjuvant, and specifically discuss the clinical outcomes of this vaccination.

## Rationales behind peptide-based cancer vaccination

Breast cancer, as a type of cancer, is predominantly consequential of genetic mutations, which progressively accumulate in number and increase in heterogenicity during the course of disease development. These accumulated mutations in breast cancer can be tumour-specific antigens (TSA), which are expressed only in cancer cells not normal cells, or tumour-associated antigens (TAA), which are expressed comparatively more in cancer cells than normal cells (Janelle et al. 2020). These antigens presented by major histocompatibility complex (MHC) molecules on antigen-presenting cells, e.g., dendritic cells, can be recognised by *T* cells to trigger cytotoxic activity against cancer cells, in the process known as cancer surveillance (Zhang et al. 2017). Therefore, with the appreciation of either TSA or TAA being competent to elicit anti-cancer immunity, peptide-based cancer vaccine has become one of the novel therapeutic approaches under development, by incorporating the epitopes derived from tumour antigens to elicit anti-cancer *T*-cell immunity (Thundimadathil 2012). Normally, the epitope consists of an 8 to 10 or 12 to 18 amino-acid-long peptide fragment of antigens that are only or more expressed in cancer cells, either TSA or TAA, and is ideally designed optimal for internalisation by dendritic cells and antigen presentation on MHC molecules, delivering CD8^+^ or CD4^+^ *T* cell activation, respectively (Hines and Marschall 2018).

However, the majority of cancer cells, including breast cancer, are capable of evolving the mechanism of “avoiding immune destruction”, as one of the emerging cancer hallmarks proposed by Hanahan and Weinberg (2011), to strive for survival and proceed on growth. To combat the tumour immune suppression, cancer vaccination also encompasses the use of adjuvants, which simultaneously incite innate immunity and enhance the antigen presentation by dendritic cells or other antigen-presenting cells, thereby aiding the *T*-cell-mediated immunity (Temizoz et al. 2016; Vermaelen 2019). Meanwhile, peptide-based vaccines regarding different tumour antigens or epitopes in breast cancer are increasingly under investigation in continuous clinical trials, where the assessment of the additional effect of adjuvants is included.

## HER2/neu-based vaccination using GM-CSF as the adjuvant

Among all peptide-based vaccines in development, one of the most common TAAs in breast cancer, HER2/neu, has undergone three phases of clinical trials (Thundimadathil 2012). HER2/neu is a kind of receptor tyrosine kinase in the HER family and is responsible for activating signalling pathways for cell differentiation, adhesion and growth (Ménard et al. 2004). Overexpression or gain-of-function mutation of HER2/neu leads to the formation of its oncogene as discovered in 15 to 30% of all breast cancers. Its close association with survival decline has promoted it to be a powerful prognostic and predictive biomarker in breast cancer, and therapies targeting the overexpressed HER2/neu peptides or function are one of the most studied breast cancer therapeutics (Iqbal and Iqbal 2014). In the context of breast cancer vaccination, three HER2/neu-derived epitopes (E75, GP2 and AE37) (Table 1) have been developed into the vaccines, to promote the immune targeting of breast-cancer specific HER2/neu peptide and accelerate cytotoxic activity against HER2/neu-expressing cancer cells.

**Table 1:** Clinical trials of HER2/neu-based vaccination for breast cancer (Carmichael et al. 2010; Clifton et al. 2017, 2020; Holmes et al. 2008; Mittendorf et al. 2014, 2016, 2019; Patel et al. 2021b; Peace et al. 2017)

HER2/neu epitopes	Study ID	Phase	Subtype	Enrolment	Eligibility	Adjuvant	Combined treatment	Outcome
E75 (KIFGSLAFL)	NCT00841399 /NCT00584789	I/II	Disease-free	195; 187 assessed	Lymph node-positive/negative, HER2/neu by Immunohistochemistry 1–3 +	GM-CSF	None	Safety and efficacy
	NCT01479244	III	Disease-free	758	Lymph node-positive, HER2/neu by Immunohistochemistry 1/2 +	GM-CSF	None	Safety; no efficacy
	NCT01570036	IIb	Disease-free	275	Lymph node-positive, HLA-A2/A3/A24/A26 + HER2/neu by Immunohistochemistry 1/2 +	GM-CSF	Trastuzumab	Safety; efficacy in triple-negative
GP2 (IISAVVGIL)	*N/A*	I	Disease-free	18	Lymph node-negative, HLA-A2 + , HER2/neu by Immunohistochemistry 1–3 +	GM-CSF	None	Safety and efficacy
	NCT00524277	II	Disease-free	180	Lymph node-positive/negative, HLA-A2 + , HER2/neu by Immunohistochemistry 1–3 +	GM-CSF	None	Safety; minimal efficacy
	NCT03014076	lb	Disease-free	17	HLA-A2/A3 + , HER2/neu-positive	GM-CSF	Trastuzumab	Safety and efficacy
	NCT00524277	IIb	*N/A*	168	Lymph node-positive/negative, HLA-A2 + , HER2/neu immunohistochemistry 1–3 +	GM-CSF	After trastuzumab	Safety and efficacy for recurrence
AE37 (GVGSPYVSRLLGICL)	*N/A*	I	Disease-free	15	Node-negative, HER immunohistochemistry 1–3 +	GM-CSF	None	Safety and efficacy
	NCT00524277	II	Disease-free	298	Node-positive/negative	GM-CSF	None	Safety; efficacy in HER2/neu-low and triple-negative

*GM-CSF* granulocyte–macrophage colony-stimulating factor, *HER2/neu* human epidermal growth factor receptor 2, *HLA* human leukocyte antigen

In addition, incorporating adjuvants in anti-HER2/neu vaccine therapy for breast cancer is a significant strategy with the intention to boost the immune response against the neoantigen exposed as the vaccine target. Surprising, existing clinical studies have revealed that GM-CSF is the only adjuvant in conjunction with HER2/neu peptide vaccines in the current breast cancer clinical trials, provided with its greater promising clinical value in treating melanoma, ovarian or prostate cancer than other adjuvants (Clive et al. 2010). GM-CSF even constitutes the sole agent for treating prostate cancer and melanoma as it is responsible for the survival and differentiation of myeloid cell progenitors and immune enhancement of cytokine secretion, aiding antigen presentation and lymphocyte-based immunity (Hamilton 2019; Lotfi et al. 2019). Therefore, this section will mainly summarise three HER/neu epitopes (E75, GP2 and AE37) in vaccines and their major clinical findings with GM-CSF immunoadjuvant.

### E75

E75, also known as Nelipepimut-S, is one of the most concerned epitopes composed of 369 to 377th amino acids (KIFGSLAFL) from the extracellular domain of HER2/neu protein. It is restricted to binding to human leukocyte antigen (HLA)-A2/A3 MHC class I molecules, and therefore, can activate cytotoxic CD8^+^ *T* cells for anti-cancer responses (Clifton et al. 2015, 2016). In a phase I/II trial (NCT00841399/NCT00584789) reported by Mittendorf et al. (2014), 195 breast cancer patients (lymph node-positive/negative, HER2/neu by Immunohistochemistry 1–3 +), who have already been treated with standard of care surgery, chemotherapy and radiation and continued to receive prescribed endocrine therapy, were enrolled and 6 withdrew consent. In the dosing regimens, 108 HLA-A2/A3 + patients were given intradermally with monthly inoculations of E75 peptide (100, 500, 1000 μg) and GM-CSF (125, 250 μg) for overall 4 or 6 months while 79 HLA-A2/A3- patients were not vaccinated as the control group. Vaccinated patients had minimal toxicities and in vitro immunological responses were observed in all groups (*p* ≤ 0.02) as indicated by delayed-type hypersensitivity (DTH). However, there is a significant improvement in the 5 year disease-free survival (DFS) in optimally dosed patients only (94.6 vs. 80.2% as the control, *p* = 0.05), and a decrease in recurrence risk by 48%.

Meanwhile, E75 is the only epitope that has accomplished the phase III breast cancer clinical trial (NCT01479244), and developed as the NeuVax™ for breast cancer therapeutic vaccination (Mittendorf et al. 2019). Similar to the clinical trial performed by Mittendorf et al. (2014), 1000 μg of E75 peptide in 250 μg of GM-CSF was monthly given to 376 breast cancer patients (lymph node-positive, HER2/neu by Immunohistochemistry 1/2 +) for 6 months, with placebo replacing E75 given to another 382 patients as the control. The most adverse events were associated with injection and did not differ between groups. No significant difference (*p* = 0.07) has been discovered in DFS at a median follow-up of 16.8 months, which was also the termination time due to futility.

In addition, another phase IIb trial (NCT01570036) has recruited and randomised 275 HLA-A2/A3/A24/A26 + disease-free patients (HER2/neu by Immunohistochemistry 1/2 +), who received trastuzumab for 1 year, with concurrently 136 vaccinated with 1000 μg of E75 plus 250 μg of GM-CSF and 139 treated with placebo plus 250 μg of GM-CSF as the control every three weeks for six times (Clifton et al. 2020). Co-treatment of vaccination and trastuzumab did not present significant toxicity. No significant improvement in DFS (89.8 vs. 83.8% as the placebo control, *p* = 0.18) after the median 25.7 months of follow-up was observed further, yet DFS was elevated in patients with triple-negative breast cancer (92.6 vs. 70.2% as the placebo control, *p* = 0.01) upon vaccination. This evidence implies the clinical efficacy of E75 with GM-CSF and trastuzumab in triple-negative breast instead of general breast cancer.

### GP2

Having the same length as E75, GP2 is the second HLA A2 + restricted epitope, which fragments from 654 to 662nd amino acids (IISAVVGIL) of HER2/neu proteins at the transmembrane domain. It is also responsible for inducing cytotoxic activity, with even greater immunogenic potential to produce a profound therapeutic effect, in comparison to E75 (Clifton et al. 2015). In response, a phase I clinical trial has enrolled 18 disease-free patients (lymph node-negative, HLA-A2 + , HER2/neu by Immunohistochemistry 1–3 +) who received 6 monthly inoculations of GP2 (100, 50, 1000 μg) with GM-CSF (125, 250 μg) (Carmichael et al. 2010). GP2 was well-tolerated and activated immunologic responses measured by DTH from 0 to 27.5 mm (*p* < 0.001) and CD8 + *T* cells from 0.8 to 1.6% (*p* < 0.001). Another phase II trial (NCT00524277) assessed the GP2 peptide vaccine efficacy in disease-free breast cancer patients (lymph node-positive/negative, HLA-A2 + , HER2/neu by Immunohistochemistry 1–3 +), in the subject to either presence or absence of 500 μg of GP2 with 125 μg of GM-CSF (Mittendorf et al. 2016). Consistently, GP2 plus GM-CSF was safe and presented a 48% decrease in recurrence rate, though no significant difference in 5 year DFS rate (90 vs 80% with GM-CSF alone, *p* = 0.08) was seen at a median follow-up of 60 months.

GP2 vaccine was also investigated along with trastuzumab. A phase Ib trial (NCT03014076) indicated that GP2 vaccine with GM-CSF and trastuzumab was obligated to trigger interferon-γ secretion (144 vs 46 secreting spots as the baseline, *p* = 0.13) indicative of immunologic responses, and as well safe in 17 disease-free, HER2/neu-high-expressing, HLA-A2/A3 + breast cancer patients (Clifton et al. 2017). Besides, the 5-year median follow-up data of a phase IIb study (NCT00524277) has indicated that 6 3/4-week-interval vaccinations of 500 μg of GP2 in 125 μg of GM-CSF improved rarely in 5 year DFS (77.1 vs. 77.6% as the placebo control, *p* = 0.9142) but dramatically in recurrence rate, which was down to 0% in breast cancer patients (HER2/neu immunohistochemistry 1–3 +) after adjuvant trastuzumab therapy (Patel et al. 2021b), and phase III trial was designed similarly (Patel et al. 2021a). In this case, GP2 plus GM-CSF may be more effective in decreasing recurrence, rather than improving 5-year DFS, even with the addition of adjuvant trastuzumab, which nearly eliminated recurrence in the phase II trial.

### AE37

AE37 is another peptide containing 776 to 790th amino acids (GVGSPYVSRLLGICL) at HER2/neu intracellular domain in the hybrid with a 4-amino-acid-long li-Key (LRMK) derived from the invariant chain (li) of MHC class II molecule (Clifton et al. 2015). Unlike E75 and GP2, AE37 can activate CD4^+^ *T* cells (Sotiriadou et al. 2006; Voutsas et al. 2007) and suppress the activity of regulatory *T* cells (Gates 2009) in preclinical studies. Subsequently, Holmes et al. (2008) conducted a phase I clinical trial that presented the minimal toxicity of the AE37 (100, 500, 1000 μg) with GM-CSF (0, 30, 125, 250 μg) in 15 disease-free breast cancer patients (node-negative, HER immunohistochemistry 1–3 +). More essentially, AE37 itself, in the absence of GM-CSF as the adjuvant, was proven to stimulate immune responses specific to HER2/neu from DTH 8.3 to 65.7 mm. Another phase II trial (NCT00524277) demonstrated improved DFS in vaccinated patients (node-positive/negative) who are HER2/neu-low-expressing at disease stage IIB/III (90 vs. 32% with GM-CSF alone, *p* = 0.02) or triple-negative (89 vs. 0% with GM-CSF alone, *p* = 0.05) (Peace et al. 2017) at a median follow-up of 25 months. Therefore, AE37 with GM-CSF may be more potent than the other two epitopes to clinically improve the DFS of HER2/neu-low-expressing patients, including those being triple-negative. This discovery may imply the underlying significance of including li-Key hybrid in structure to target CD4^+^ T cell immunity, which may be more essential for rendering AE37 effectiveness.

## Discussion

Current HER2/neu-based vaccine trials are mostly associated with disease-free breast cancer patients who have completed the standard therapies, such as surgery, radiotherapy or chemotherapy (Carmichael et al. 2010; Clifton et al. 2017, 2020; Holmes et al. 2008; Mittendorf et al. 2014, 2016, 2019; Patel et al. 2021a, b; Peace et al. 2017). This is because GM-CSF, as the immunoadjuvant generally used in almost all HER2/neu-based clinical trials, degrades rapidly, and therefore, is more suitable for vaccines used to prevent future recurrence of disease when the disease burden is low or transiently none (Schneble et al. 2014). Therefore, HER2/neu-based vaccines can entail the development into a powerful therapeutic tool in the coalescence with adjuvants which can persist for long-term immune stimulation.

Trials for E75 and GP2 were limited to eligible populations who are HLA A2 and/or A3 + (Carmichael et al. 2010; Clifton et al. 2017, 2020; Mittendorf et al. 2014, 2016, 2019; Patel et al. 2021a, b), whilst there is no such constrain for AE37 (Holmes et al. 2008; Peace et al. 2017), as HLA A molecules form the major group of MHC class I molecules, which are targeted by E75 and GP2. Therefore, the use of AE37 vaccines to treat breast cancer patients may be less restricted in terms of HLA A subtypes, compared to E75 and GP2, but it remains arguable for AE37 trials which did not include the HLA typing for MHC class II molecules for population selection.

Although the phase I/II trial (NCT00841399/NCT00584789) for E75 with GM-CSF has discovered the statistical significance in immune response and 5-year DFS, this is also arguable for assigning HLA A2/A3- patients as the control groups (Mittendorf et al. 2014). HLA A2/A3 status may not be a direct indicator to represent the presence or absence of the vaccine effect, even though E75 was preclinically analysed to be HLA A2/A3-restricted (Clifton et al. 2015). Therefore, inconsistent with the phase III trial (NCT01479244) where the control replaced E75 with a placebo, the significant improvement in DFS no longer emerged (Mittendorf et al. 2019). Moreover, no significance in 5 year DFS also happened to the phase II trial (NCT00524277) with GP2 and GM-CSF, though they have previously presented immunological response in phase I study (Carmichael et al. 2010; Mittendorf et al. 2016).

Nevertheless, the E75 vaccine plus GM-CSF (NCT01570036) with concurrent treatment of trastuzumab indicated DFS improvement in triple-negative patients at a median of 25.7 months (Clifton et al. 2020). This study also simultaneously strengthens the advice for the use of HER/neu-based vaccine in HER2/neu-negative patients. Besides, HER2/neu-based vaccines combined with other anti-HER2/neu therapies like trastuzumab may convey greater immune responses, and trastuzumab alone with GM-CSF did not aid in the immune activation in HER/neu-negative patients. By blocking the function of HER2/neu protein at its extracellular domain to decelerate cell proliferation and expedite apoptosis, trastuzumab can serve with antibody-dependent immunomodulation by recruiting CD8^+^ *T* cells for cytotoxic immunity (Hudis 2007). Potentially, trastuzumab may as well render such clinical improvement by destroying HER2-expressing cancer cells, which expose various intracellular antigenic peptide targets for immune recognition (Maximiano et al. 2016). Therefore, the addition of a HER2/neu-based vaccine may boost the anti-HER2/neu effect of trastuzumab especially when the expression of HER2/neu is considered to be low. On the other hand, given that trastuzumab adjuvant to the GP2 vaccine and GM-CSF (NCT00524277) gave a better recurrence rate only (Patel et al. 2021b), whether trastuzumab can aid the immune targeting of vaccine antigens to unleash the therapeutic efficacy remains unknown but is essential for future investigation.

In accordance with it, AE37 with GM-CSF illustrated vivid progress on 25-month DFS with HER2/neu-low-expressing or triple-negative patients in its phase II trial (NCT00524277) (Peace et al. 2017), implying that epitope involvement of li-Key hybrid in AE37 can be more effectively recognised by CD4^+^ T lymphocytes for immune stimulation, in comparison to E75 and GP2 which are epitopes alone. Nevertheless, given that li-Key peptides bind at the allosteric site distant from the antigen binding site and expedite the antigen presentation by MHC class II molecules, antigenic epitope linked with li-Key hybrid significantly enhances the affinity of the epitope being bound to MHC class II molecule by 250-fold and increases antigen recognition (Humphreys et al. 2000), leading to *T* cell responses clinically (Peace et al. 2017). The potential reason may be underlined by the li-Key-peptide-driven structural conformation on MHC class II molecules, in favour of the binding to antigen and formation of the MHC-peptide-*T*-cell receptor complex. This finding has highlighted the vaccine design to enhance the effectiveness of vaccines by adding the epitope to a hybrid structure that regulates antigen presentation on MHC class molecules.

On the other hand, the success of AE37 in triggering immunity and prolonging DFS in HER2/neu-low-expressing or triple-negative patients (NCT00524277) may be due to the absence of HER2/neu-associated immune inhibition. In brief, the HER2 peptide vaccine may not alleviate the medical conditions of HER2/neu-high-expressing patients, as their immune system has been defective in responses to high HER/neu expression, and therefore, tolerate the survival and growth of cancer cells. Comparatively, although HER2/neu-low-expressing or triple-negative patients are “negative” for HER2/neu expression but expressed at the non-detectable or negligible level, increasing the exposure to HER2/neu epitope via vaccination with li-Key hybrid can promote the immune recognition of these cancer cells as long as they express some but a few HER2/neu proteins. Using anti-HER2/neu antibodies like trastuzumab to supplement the AE37 vaccine is as well more likely to turn “HER2/neu-negative” into “HER2/neu-positive” for immune detection but such a proposal requires further confirmation.

Another major difference in clinical outcomes between these three vaccines originated from their MHC restriction. Attributed to AE37 being MHC II-responsive, GM-CSF may boost the AE37-mediated immunity by upregulating the expression of MHC class II molecules, though AE37 alone can produce immunological responses (Hamilton 2019; Lotfi et al. 2019). It suggested that GM-CSF may not be the most decant adjuvant for AE37 still or the other two epitope vaccines. In addition, E75 and GP2 utilise MHC class I molecule signalling that is primarily triggered by endogenous peptides in the cytosol, whilst AE37, for its li-Key hybrid, activates MHC class II molecule signalling as an exogenous antigen (Murphy and Weaver 2017). Therefore, E75 and GP2 may be more problematic to trigger the endogenous MHC class I pathway as vaccination delivers the exogenous source, unless cross-presentation takes place to alert the MHC class I pathway for exogenously sourced antigens (Murphy and Weaver 2017). Addressing the clinical trials aforementioned, GM-CSF is only related by increasing the formation of antigen-presenting cells and *T* cell clones as essential components in the process of cross-presentation, and therefore, is the universal adjuvant for application. In this case, to take advantage for future study design, an adjuvant with cross-presentation enhancing function, e.g., Toll-like receptor 4 agonists that have been licensed clinically as vaccines adjuvants for human papillomavirus and melanoma for its enhancement on processes associated with cross-presentation, can be co-administrated with either E75 or GP2 vaccine to aid the loading of exogenous antigens to MHC I class molecule and CD8^+^ T lymphocyte activation (Ho et al. 2018; Lee and Suresh 2022).

From the perspective of safety, all three HER2/neu peptides were well-tolerated in clinical trials. In detail, both E75 (Mittendorf et al. 2014, 2019) and GP2 (Carmichael et al. 2010; Mittendorf et al. 2016) presented mostly grade 1 or 2 toxicities that are local (injection-related erythema, induration or pruritis) and system (fatigue, headache influenza-like illness, arthralgia or myalgias). Although grade 3 adverse events occurred in 1 to 2% of either phase III trial of E75 the peptide (Mittendorf et al. 2019) or phase II trial of the GP2 peptide (Mittendorf et al. 2016) still, there was no significant difference in toxicity when compared to the placebo or control groups. Similarly, the addition of trastuzumab could not contribute to any control-compared toxicity difference and was also associated with mostly grade 1 and 2 toxicities, including induration and pruritis (Clifton et al. 2017, 2020; Patel et al. 2021b). Despite the lack of a detailed toxicity profile for the AE37 trial in phase II (Peace et al. 2017), its phase I trial has illustrated that the maximum symptoms were mainly grade 1 for systemic toxicities and grade 2 for local toxicities (Holmes et al. 2008), providing the promising insights into the safety of AE37.

Overall, the HER2/neu peptide vaccine for breast cancer remains a novel and feasible anti-cancer strategy with only a few clinical outcomes being discovered and the majority of the completed clinical trials are concerned about the disease recurrence instead of curing cancer. Learning from the current experience, the attempts to include a hybrid with an epitope in vaccination, optimise adjuvants with more relevance to the epitope-mediated immunity, combine with anti-HER2/neu therapies and stratify eligible patients who express low HER2/neu are the most remarkable steps for approaching the future therapeutic development for breast cancer.

## Data Availability

No datasets have been generated in this review, so no data availability statement is included.
